# Draft genome sequence of *Erwinia amylovora* strain XJ14 originated from Hawthorn in China

**DOI:** 10.1128/mra.00440-26

**Published:** 2026-06-03

**Authors:** Yuqiang Zhao, Jianan Wang, Yanli Tian, Baishi Hu

**Affiliations:** 1 Institute of Botany, Jiangsu Province and Chinese Academy of Sciences (Nanjing Botanical Garden Mem. Sun Yat-sen) 101674 , Nanjing, China; 2 College of Plant Protection and Key Laboratory of Integrated Management of Crop Diseases and Pests, Ministry of Education, Nanjing Agricultural University 70578, Nanjing, China; 3 Key Laboratory of Plant Quarantine Pests Monitoring and Control, Ministry of Agriculture and Rural Affairs, Nanjing, China; 4 Xinjiang Key Laboratory of Agricultural Biosafety, Urumqi, China; 5 College of Agriculture, Xinjiang Agricultural University 117840, Urumqi, China; Rochester Institute of Technology, Rochester, New York, USA

**Keywords:** *Erwinia amylovora*, fire blight, China, hawthorn

## Abstract

Fire blight, caused by *Erwinia amylovora*, threatens global apple and pear production and was first reported in China in 2016. We report the draft genome sequence of *E. amylovora* XJ14, a hawthorn isolate from Altay, Xinjiang, China’s first *E. amylovora* genome from this host.

## ANNOUNCEMENT

*Erwinia amylovora*, the causal agent of fire blight, is an important agricultural plant pathogen infecting rosaceous plants worldwide. The symptoms of fire blight were first documented in the USA in 1793, and the disease has since spread globally via human-mediated movement of infected plant material (1). Fire blight in China was first discovered in Yili Kazak Autonomous Prefecture and Bayingolin Mongolian Autonomous Prefecture, Xinjiang Province, in 2016, affecting pear, apple, hawthorn, crabapple, and quince (2).

Herein, we report the draft genome sequence of an *E. amylovora* strain XJ14 isolated from symptomatic hawthorn in Altay, Xinjiang Province (48.084319° N, 86.414479° E) in 2018. Because of their excellent pollution resistance, cold resistance, drought tolerance, tolerance to thin soil, and ornamental value, hawthorns are widely used as street trees.

Symptomatic tissues were surface-sterilized with 75% ethanol. Small pieces of leaves from the lesion margins were ground in sterile water, streaked onto Luria–Bertani medium, and incubated at 28°C for 36–48 h. Single colonies were purified by subculturing. Isolate XJ14 was positively identified as *E. amylovora* by PCR using specific primer pairs p29A/B and Koch’s postulates. Genomic DNA was extracted from 2 mL of overnight culture using the TIANamp Bacteria DNA Kit (Tiangen Biotech, Beijing, China).

Whole-genome sequencing was conducted using an Illumina HiSeq 4000 system (Illumina, San Diego, CA, USA) using 150 bp paired-end libraries with insert sizes of 270 and 6,000 bp at the Beijing Genomics Institute (Shenzhen, China). Raw reads were filtered to remove low-quality sequences (Phred score ≤20), adapter contamination, duplicates, and reads with >10% ambiguous bases, yielding 14,758,119 clean reads from 17,635,994 raw reads. *De novo* assembly was conducted using SOAPdenovo v1.05 with default parameters (3). The final assembly comprised 56 contigs (3,809,165 bp; N50: 267,956 bp) and 24 scaffolds (3,841,238 bp; N50: 3,788,872 bp), with a GC content of 53.53%, an average sequencing depth of 43.09×, and genome coverage of 99.16% (≥1×, 97.85% covered by ≥5 reads).

Protein-coding genes were predicted with Glimmer v3.02 (4). Non-coding RNAs were annotated using tRNAscan-SE v1.3.1 (−B bacterial mode) (5) and RNAmmer v1.2 (−S bac) (6). Tandem repeats, prophages, and CRISPR elements were identified with Tandem Repeat Finder v4.04 (parameters: 2 7 7 80 10 50 2000 -d -h) (7), PHAST v2013.03 (8), and CRISPRFinder v0.4 (9), respectively. Functional annotation was performed using BLAST+ v2.2.28 (e-value cutoff 1e-5) against seven databases, including KEGG, COG, NR, Swiss-Prot, GO, TrEMBL, and EggNOG. Default parameters were used except where otherwise noted. In total, 3,835 protein-coding genes, 73 tRNAs, and 10 rRNAs were identified.

Average nucleotide identity (ANI) and digital DNA–DNA hybridization (dDDH) were calculated using OrthoANI v0.93.1 (10) and GGDC v3.0 (11), respectively. Strain XJ14 shared 99.93%–99.95% ANI and >70% dDDH with *E. amylovora* type strains, confirming its species identity (12) (Table 1).

**TABLE 1:** ANI and dDDH values between strain XJ14 and members of *E. amylovora^a^*

	CFBP1430	ATCC49946	Ea1189
ANI (%)	99.95	99.93	99.95
dDDH (%)	99.9	99.7	99.8

^*a*^ ANI, average nucleotide identity; dDDH, digital DNA–DNA hybridization.

## Data Availability

The draft genome of XJ14 has been deposited at GenBank under BioProject accession number PRJNA1087690, BioSample accession number SAMN40453203, and GenBank accession number JBBJYR000000000.
